# Evidence-Based Conceptual Collection of Methods for Spatial Epidemiology and Analysis to Enhance Cancer Surveillance and Public Health

**DOI:** 10.3390/ijerph191912765

**Published:** 2022-10-06

**Authors:** Dimitra Sifaki-Pistolla, Vasiliki Eirini Chatzea, Elpiniki Frouzi, Enkeleint A. Mechili, Georgia Pistolla, George Nikiforidis, Vassilis Georgoulias, Christos Lionis, Nikos Tzanakis

**Affiliations:** 1 Cancer Registry of Crete, School of Medicine, University of Crete, 71003 Heraklion, Greece; 2 Hellenic Mediterranean University, 71410 Heraklion, Greece

**Keywords:** spatial epidemiology, spatial statistics, cancer surveillance, environmental epidemiology, global health, chronic diseases, respiratory diseases, cancer

## Abstract

(1) Background: Although spatial statistics are often used by cancer epidemiologists, there is not yet an established collection of methods to serve their needs. We aimed to develop an evidence-based cancer-oriented conceptual collection of methods for spatial analysis; (2) Methods: A triangulation of approaches was used; literature review, consensus meetings (expert panel), and testing the selected methods on “training” databases. The literature review was conducted in three databases. This approach guided the development of a collection of methods that was subsequently commented on by the expert panel and tested on “training data” of cancer cases obtained from the Cancer Registry of Crete based on three epidemiological scenarios: (a) low prevalence cancers, (b) high prevalence cancers, (c) cancer and risk factors; (3) Results: The final spatial epidemiology conceptual collection of methods covered: data preparation/testing randomness, data protection, mapping/visualizing, geographic correlation studies, clustering/surveillance, integration of cancer data with socio-economic, clinical and environmental factors. Some of the tests/techniques included in the conceptual collection of methods were: buffer and proximity analysis, exploratory spatial analysis and others. All suggested that statistical models were found to fit well (R2 = 0.72–0.96) in “training data”; Conclusions: The proposed conceptual collection of methods provides public health professionals with a useful methodological framework along with recommendations for assessing diverse research questions of global health.

## 1. Introduction

Modern societies are increasingly facing the direct and indirect effects of the high burden of the non-communicable diseases (NCDs), such as cancer, mental health disorders, obesity, diabetes, cardiovascular and cerebrovascular disease, chronic obstructive pulmonary disease (COPD), and arthritis [1]. During recent years, cancer has been considered to be among the major epidemics in many countries, with trachea, bronchus and lung cancer having caused 1,694,600 deaths in 2015, worldwide [2]. The global epidemiologic and demographic transitions indicate an ever-increasing cancer burden, with over 20 million new cancer cases expected annually prior to 2025; especially in low-middle income countries (LMIC) [3].

One of the major challenges that cancer epidemiologists and public health researchers are tackling is how they could form and suggest effective and comprehensive measures to establish cancer control programs [4]. Currently, the nature of cancer control is changing, with an increasing emphasis on risk factor controlling and prevention, early diagnosis, as well as patient experience during and after treatment [5]. Therefore, public health professionals and cancer researchers need to think globally but act locally based on reliable findings. In other words, they have to correctly identify causal relationships, risk factors and populations at risk as well as to predict future dispersion of diseases at the local level [6,7]. Towards this direction, they often employ several statistical techniques with the aim to optimize quality of cancer data and analysis, improve methodologies or tools, enhance results and conclusions [8,9].

Recent public health approaches stress the importance of analyzing epidemiological data with respect to the individual characteristics and explore the under-studied phenomenon holistically. This is strongly related not only to the main epidemiological principles (disease, person, place, time) [10], but also to the wider concept of “space” [11,12]. “Space” does not refer to the geographical location (e.g., location of residence) alone, it includes environmental conditions and exposures, societal framework, culture, religion, economy, habits, human interactions and any phenomenon that occurs on earth or any sub-entity, such as human. These phenomena occur in the so called “real world” or more correctly the “spacetime” [12], while they refer to quantitative and qualitative approaches that are met in the philosophy of the Geographical Information Systems (GIS).

GIS and spatial statistics are rapidly growing in cancer epidemiology and public health research [7,13,14,15]. They are accredited by public health professionals and cancer registries as powerful tools, since they can play a critical role in determining major public health questions such as “where”, “when”, “how” and “who”, and provide a wide range of statistical methods and tools [12]. Several GIS tools are employed by public health researchers to respond to questions such as “Where the disease started?”, “How and to which direction is the disease spreading?”, “Where are sources of air pollution?”, “Which are the causal or risk factors of disease per geographical region?” and many more. Public health researchers use GIS in diverse research, planning and disease management projects to gain knowledge on disease mapping, identification of disparities and inequalities and the determination of where new interventions and policies should be focused. Most of the public health-related studies and the vast majority of the cancer studies employ GIS mainly for proximity measures. In addition, cancer clustering or simple distribution mapping of cancer rates are among the predominant spatial approaches [16]. Contrary to that, aggregation tools (spatial smoothing and interpolation techniques), space-time clustering and spatial regression are still missing from the literature although they seem to have expanded steadily during recent years [12,15]. The gap of technical and theoretical knowledge between these two different sciences, epidemiology and geography, may be among the main barriers to the exploitation of all different geo-epidemiological methodologies in cancer statistics. This vacuum should be filled in order to enhance public health measures towards effective cancer control. Solid epidemiological data and validated statistical methods of analysis are required to capture cancer particularities and contribute to reliable decision making.

Therefore, the development of a cancer-oriented conceptual collection of methods for spatial epidemiology that could be utilized by public health professionals, epidemiologists, other cancer researchers and cancer registries could be the cornerstone towards filling the gap and enhancing public health. Within the context of this manuscript, the term “toolkit” refers to a set of statistical tests/techniques and spatial analysis methodologies designed to be used together or for a particular purpose when managing and analyzing cancer epidemiological data. The current study aims to propose an evidence-based cancer-oriented toolkit for spatial epidemiology and analysis by synthesizing the existing knowledge from the literature, the experts’ opinion and measurable indicators. In addition, a grouping of the statistical tests/techniques, included in the toolkit according to the type of cancer data is among the main objectives of this study.

## 2. Materials and Methods

To assess the aim and objective of this study, a triangulation of approaches was performed: (a) literature review, (b) consensus meeting (expert panel), (c) testing the selected methods on real “training” databases. The outcomes of the literature review were used to form the first version of the toolkit which was subsequently appraised and approved within a consensus meeting of a group of experts. Upon this meeting, a second version of the toolkit was developed and further tested using real data in “training” databases in order to be validated. Three different scenarios were also formed and utilized for these data “training” procedures: (a) low prevalence cancers, (b) high prevalence cancers, and (c) cancer data jointly with demographic, socio-economic, clinical and environmental factors. When data are derived from “big-databases” of more than two decades of data, the categorization is proposed as follows; low prevalence cancers: less than 3 new cases/100,000/year and high prevalence cancers: more than 3 new cases/100,000/year. When data are collected for occasions or for a specific time period, then power analysis is considered essential prior analysis and selection of a research scenario. Further information on the methodological framework and processes is provided in the following sections. 

### 2.1. Literature Review

The literature review followed the majority of the principles of a systematic review based on the PRISMA guidelines [17]. The review and the critical appraisal were conducted in order to serve the following research questions: (a) “Are there any cancer or chronic disease oriented toolkits or guidebooks for spatial epidemiology and analysis?”, (b) “Which are the main functions, or dimensions or sets of methodological procedures for spatial epidemiology and analysis on cancer data?” and (c) “Which tests or particular techniques are appropriate for each type of cancer data or spatial epidemiological scenario during the statistical analysis?”.

The systematic review was conducted in three databases; PubMed, the Cochrane Library, and Google Scholar, while all records were evaluated by four independent reviewers *(V.E.C.*, *E.F., E.A.M., G.N.) and a fifth GIS expert (D.S.-P.) approved the final evaluation and helped with reconciliation*. Methodological approach and core findings of each study were reviewed based on the inclusion and exclusion criteria [18]. Specifically, several inclusion and exclusion criteria were adopted according to the PICO Framework [P: Populations, I: Interventions, C: Comparison, O: Outcomes] which is used extensively in the Cochrane Collaboration [19]. These basic components (PICO) were examined comprehensively when the inclusion criteria were applied. Then, exclusion criteria were set, with clarity and transparency, to justify the reason why some records were excluded from the study [20]. The main key words, the core algorithm and the inclusion and exclusion criteria of the search are presented in the following table (Table 1). 

**Figure 1:**
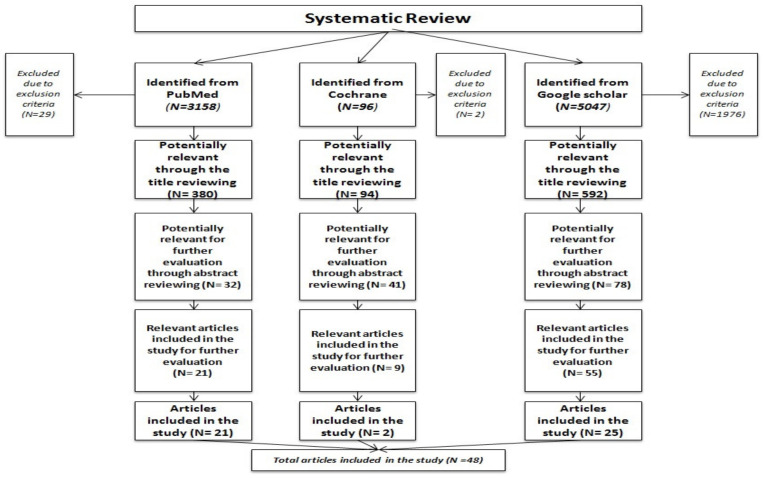
Flow chart of eligible assessment and review process.

The eligibility assessment and the review process of the records were performed separately by four independent reviewers according to the stages demonstrated in Figure 1. Potential disagreements were resolved by consensus. At first, the exported records of the three databases were combined and duplicates were removed. Not applicable records according to the exclusion criteria were also removed at this stage, in case they had not been automatically excluded by filters performance. Then, records were reviewed only by title and those that indicated ineligibility were removed. The included records were reviewed by abstract and those that were relevant for further evaluation were reviewed by full text. Finally, the eligible records were included in the study and were also used for the critical appraisal process.

After the systematic review was completed, no toolkit or guidebook was identified in the 48 included studies, therefore the authors focused on articles assessing or comparing two different statistical methodologies/techniques or utilizing such methods on cancer or chronic disease data. These studies were critically appraised by the four independent reviewers, while the fifth reviewer (D.S.-P.) was responsible for “translating” the main outcomes into a logic-model (formulation of the first draft of the toolkit).

### 2.2. Consensus Meeting

The above systematic approach guided the development of the first version of the toolkit that was subsequently appraised within an online expert panel that was organized by the first author. Ten experts of diverse scientific backgrounds (3 public health researchers/epidemiologists with focus on cancer surveillance and control, 4 GIS experts, 2 mathematicians/geographers, 1 medical oncologist) were invited and responded positively. Three rounds of discussion and commentary on the tool-kit were followed. Round 1 included the provision of general comments and feedback on the developed toolkit as well as additional input (e.g., papers, books and grey literature) that could be merged in the tool-kit. The second round aimed to appraise the revised version of the toolkit and evaluate/assess each one of its functions using a 5-point Likert scale (1. Strongly disagree, 2. Disagree, 3. Neither disagree nor agree, 4. Agree, 5. Strongly agree). The third round focused on the final approval of the toolkit, which reached a consensus of 99% agreement.

### 2.3. “Training” Data—Validation of the Toolkit

The toolkit, as developed upon the systematic review and the critical appraisal by the experts, was cross-validated using measurable quantitative indicators. In particular, it was tested in terms of validity and fitness of each suggested procedure/test using real cancer data in three different “training datasets”. “Training datasets” were selected based on three scenarios (low prevalence cancers (oral cancer), high prevalence cancers (colorectal cancer), and cancer data jointly with demographic, socio-economic, clinical and environmental factors (lung cancer as dependent variable; environmental exposures, gender, age, occupation, place of residence, clinical stage, co-morbidities, family cancer history, smoking status and alcohol consumption as covariates)). The environmental exposures in Scenario C included PM2.5, between 2.5 μm and 10 μm (PM2.5–10), PM10, PM2.5 absorbance (black carbon measure), nitrogen dioxide (NO2) and nitrogen oxides (NOx). All data were obtained from the Cancer Registry of Crete’s big-database for the period 1992–2013. All statistical tests/techniques included in the toolkit were performed separately for each one of the three scenarios, while R square test was estimated in order to assess the overall fitness per test/technique (summarizes the discrepancy between observed values and the values expected under the model in question). This validation process was carried out in STATA and ArcMap 10.3.1, while all tests were performed at 0.05 level of significance.

## 3. Results

### 3.1. Core Framework of the Toolkit

The final toolkit as developed (literature review and expert panel) and tested (“training data” procedures) was disease-adjusted (reliable for analyzing cancer data according to the previously mentioned measurable indicators). Furthermore, three major functions were proposed (Table 2) to cover the whole spectrum of spatial epidemiology: (a) data management and adjustment pre-processes, (b) mapping data, (c) spatial/spatio-temporal analysis. These functions consist of several processes that include: data preparation and testing for randomness, data protection, mapping and visualizing, geographic correlation studies, clustering and surveillance, integration of cancer data with demographic, socio-economic, clinical and environmental factors. 

### 3.2. Core Framework of the Toolkit Validated Sets of Methodologies According to the Scenarios

**Figure 2:**
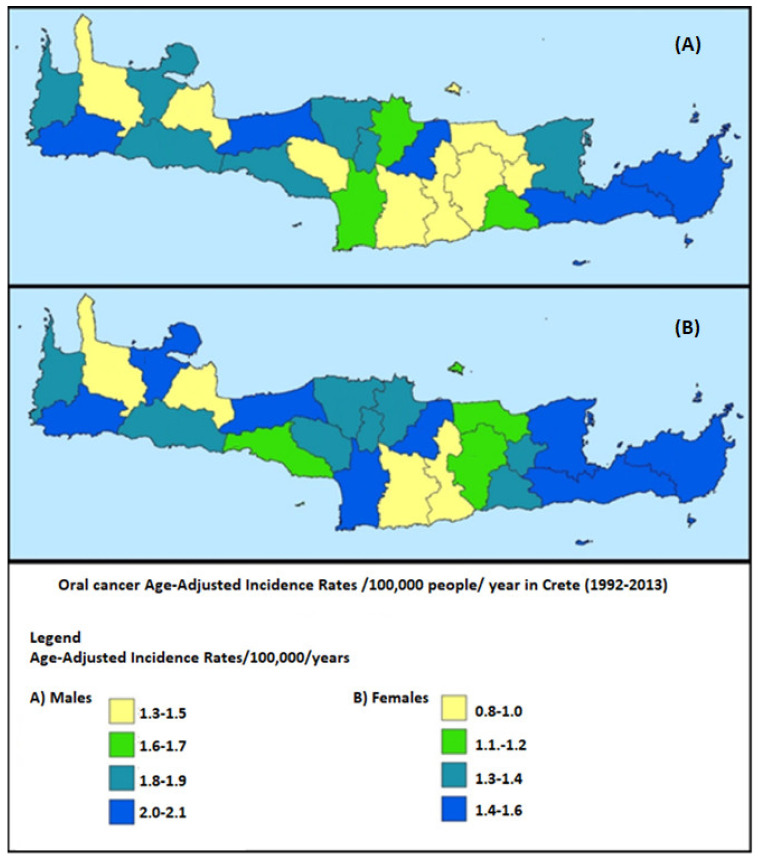
Geographical distribution of oral cancer Age-Standardized Incidence Rates/100,000/year in Crete, (Source: Cancer Registry of Crete).

**Figure 3:**
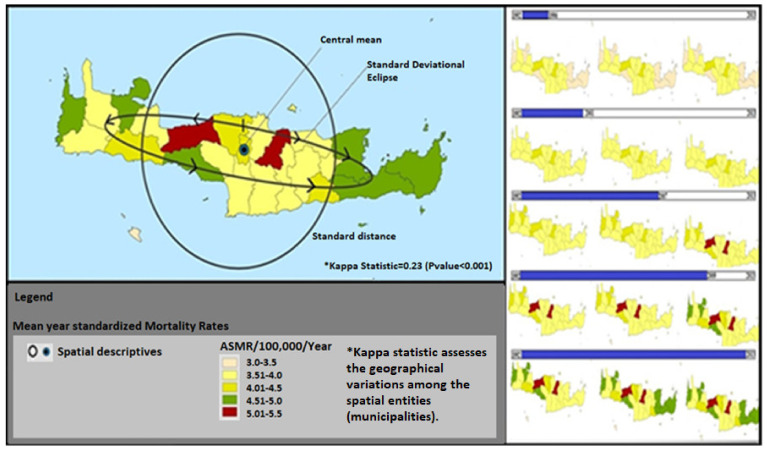
Geographical distribution and temporal trends of colorectal Age-Standardized Mortality Rates/100,000/year in Crete, (Source: Cancer Registry of Crete).

**Figure 4:**
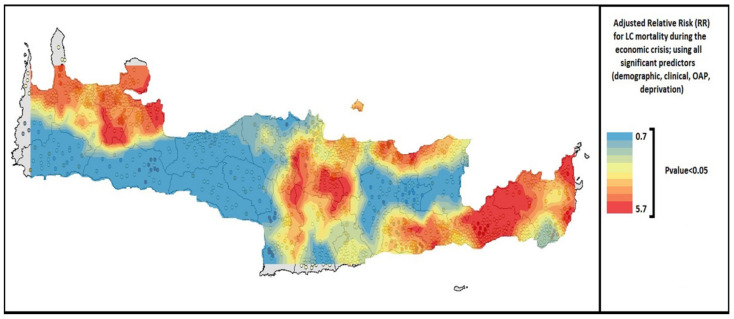
Lung cancer Relative Risk (RR) for mortality using all significant predictors [14].

All suggested statistical models that were proposed by the toolkit were validated and found to fit well (R2 = 0.72–0.96) in cancer “training data”. Good fitness of the model was determined at R2 > 0.7 and referred to the extent that each statistical procedure explained the data well and reliably; (a) low prevalence cancers (R2 = 0.72–0.85), (b) high prevalence cancers (R2 = 0.81–0.96), (c) cancer data jointly with demographic, socio-economic, clinical and environmental factors (R2 = 0.78–0.93). Table S1 (Supplementary Material) explicitly presents the steps and specific statistics tests per process, while it clarifies which test is appropriate for each type of research scenario (low or high prevalence cancers, or linked data). The main proposed methods (steps of analysis or statistical tests) included in the toolkit were: aggregation techniques for data protection, overlaying techniques, buffer and proximity analysis, exploratory spatial analysis, spatial and spatio-temporal statistics and modeling, spatial interaction models, spatial diffusion, multi-criteria analysis or regression models (e.g., Bayesians regression, Geographically Weighted Regression or the Ordinary Least Squares) and smoothing techniques that enhance health data in large databases and improve outcomes. Furthermore, three applications are provided as representative examples per cancer scenario in the following figures (Figure 2, Figure 3 and Figure 4) of Crete. 

Scenario A: In low prevalence cancers such as the oral cancer (ICD10 O: C00-C06, C09-C10, C12-C14), aggregation of the new cases at a larger geographical area (e.g., municipalities in this example) was performed to avoid stigmatizing smaller regions or cancer individuals and to manage any population differences/variations. Crete has a population of 590,000 permanent residents and is administratively grouped in 23 municipalities. In addition, local clustering tests were used but no further hot spots or regression analysis was attempted due to the small number of cancer cases per geographical unit of analysis (i.e., municipalities). Conventional regression models were utilized with an adjustment to place of residence if needed based on the data.

Scenario B: In high prevalence cancers such as colorectal cancer (ICD10 O: C18-C20), both aggregated rates and point data (according to place of residence) were used. This is suggested especially in long time-series or big-databases of more than 1000 cases. All types of spatial descriptive statistics including the standard directional ellipse were attempted. Spatio-temporal distribution and analysis were also performed in the current example. In addition, further analysis using a Bayesian regression model was conducted to identify the high-risk areas. Prior regression, spatial autocorrelation models were used to assess the randomness or clustered pattern of the under-studied cancer.

Scenario C: Lung cancer (ICD10 O: C33-C34) mortality in relation to exposure to outdoor air pollution and other risk factors (including demographic and clinical characteristics, tobacco and alcohol consumption) was used in this scenario. In addition to the basic descriptive statistics, disease clustering and hot spots analysis, further analysis was attempted; including the performance of a Bayesian multivariate regression model to assess the RR for dying from LC and testing for several exposures/risk factors. Furthermore, after identifying lung cancer predictors, projected RRs were estimated through Bayesian projection models.

## 4. Discussion

The current study managed to meet its objectives by collecting and grouping the most reliable spatial statistical techniques available in the literature and synthesize them in the form of a toolkit addressing public health professionals’ needs. This toolkit was further commented on and endorsed by the participating experts, while it was found to have good levels of fitness to cancer data. Three selected examples of such applications were provided to demonstrate visually some of the suggested methodologies, rather than to present specific results and findings.

Space and place have been key dimensions of public health and epidemiological research since its origins, yet sophisticated spatial methods have been relatively slow to trickle into cancer research [21], most probably due to disciplinary focus and training [22]. Although there is a myriad of individual tools (e.g., books, handbooks, software, instructions), as well as studies that employ simple or advanced spatial statistics in public health, there are always limitations and barriers towards the optimum utilization of these techniques. This is due to the lack of linkage between the public health research questions/objectives with the mathematical/statistical terms of each methodology [21,23,24,25]. The current study managed to develop a set of evidence-based methodologies that could be used as geo-epidemiological toolkit by public health and epidemiology researchers as well as by cancer data analysts. Responding to the objectives of this study, the toolkit was validated for cancer or chronic disease data and it could be utilized in tandem with other conventional statistical methods of analysis.

Several methodologies are suggested in the toolkit along with specific statistical tests and procedures, while they are linked with the type of cancer data that are appropriate for (e.g., low or high prevalence cancers, cancer data in relation with other types of factors). Similarly to other studies in the literature, spatial clusters [24] and spatial regression models [26] were among the main statistical processes that were included in this evidence-based toolkit. In addition to that, the current study linked these methods to specific cancer scenarios. For instance, methods of spatial clusters are the most common tool for assessing nonrandom spatial patterns of cancer data. A wide range of statistical methods have been developed to determine if disease clusters are of sufficient geographic size and concentration to have not occurred by chance [27]. The suggested global clustering tests evaluate without pointing out the specific locations of clusters, contrary to the local clustering tests that specify clusters of prefixed points.

Furthermore, most of the global cluster tests are fitted well when public health professionals attempt comparisons per country or larger geographical areas, while different test are available for smaller or larger sample sizes (e.g., low or high prevalence cancers) [28]. Local clustering tests have different substantive interpretations and detection methods, while several limitations regarding statistical power have to be overcome by the cancer researchers. Particularly, availability of cancer cases (e.g., high prevalence cancers), high variability in the background population density, multiple testing, and size and shape of cluster windows are some of the main limitations that if managed well could lead to valuable outcomes [29]. For instance, the study of Song and Kulldorff suggests the use of Tango’s MEET, which seems to have the best statistical power, while it adjusts for multiple testing and has the added value of evaluating both spatial autocorrelation and spatial heterogeneity [30]. In addition, if big-data are available in cancer epidemiological studies (e.g., in cohorts or from cancer registries), space-time clustering techniques are the predominant approach to assess the real trends of cancer. Most studies recommend the use of a global space-time Knox technique [28,31] or Diggle’s global space-time K-function [24,32,33].

According to the literature, detection of spatial patterns through the spatial regression models can lead even closer to the investigation of the underlying effects of space and other risk factors [34,35]. Many spatial regression models focus on the special parameter, but they are often parameterized in order to include covariate data which are needed for assessing the multi-variable effects of a wide range of factors that may cause or impact the observable patterns [35,36]. Bayesian spatial regression models are appropriate for estimating the effects of potential risk factors related to cancer-related outcomes [37]. The Besag York and Molliè (BYM) model is among the most reliable Bayesian approaches since it includes fixed covariates along with the random effects (fitting well even in low prevalence cancers [36,37]).

### Strengths and Limitations

GIS and spatial statistics, like every statistical approach, may hide limitations that could lead to pitfalls during the analysis [38]. Therefore, every public health or cancer researcher should always question the results before the final interpretation. The current study proposed a toolkit that may not cover each single available statistical procedure, yet it managed to capture the core ones and test them for statistical fitness. In addition, a possible limitation may yield in the fact that each cancer dataset differs and has unique characteristics that should always be considered prior analysis.

The three main scenarios selected within the context of this study attempted to shed light on the most common research scenarios in public health and cancer epidemiology. To our knowledge, this study is the first to propose an evidence-based set of cancer-adjusted methodologies for spatial analysis in public health. The fact that the authors followed a triangulation approach to develop and validate this toolkit is the major strength of the current study. The literature review offered an overview of the existing knowledge, while the consensus panel enriched this effort and qualitatively evaluated the toolkit. Additionally, the “training” data procedures cross-validated the reliability of the toolkit by utilizing quantitative measures on real cancer datasets.

## 5. Conclusions

The present study offered an inventory of spatial epidemiology techniques, while it documented the most reliable and well-fitted statistical techniques for analyzing cancer data in the form of a toolkit. The proposed toolkit conveyed a new and complete methodological framework along with evidence-based recommendations on spatial statistics and tools for assessing different types of research questions set in public health research. It was proved to be well tailored for cancer data; therefore, it is expected to enable public health professionals and analysts and enhance epidemiologic findings that will be later used to boost public health policies on cancer surveillance and control. The Cancer Registry of Crete has already adopted this toolkit for performing original public health research studies, while it strongly suggests this toolkit to other cancer registries and public health institutions. Such a toolkit could contribute to assessing and responding to major research questions of global health, such as geographical disparities, socio-economic and spatial risk factors and others.

## Figures and Tables

**Table 1:** Inclusion and exclusion criteria of the systematic review.

Main Key Words	
“spatial analysis”, “spatial statistics”, “geo-spatial analysis”, “spatial epidemiology”, “public health”, “cancer”, “malignant neoplasms”, “malignant neoplasms of the respiratory system”, “cancer burden”, “cancer epidemiology”, “cancer statistics”, “chronic disease”, “biostatistical analysis”, “data management”, Geographical Information Systems”, “GIS”, “geo-data”, “geo-location”, “location”, “place”, “time”, “longitudinal”, “methodologies”, “outcomes”, “autocorrelation”, “interpolation”, “prediction”, “clusters” and “spatial dependency”	
**Core algorithm**	
(“tool-kit” or “guidebook” or “handbook” or “set of methodologies”) and (“malignant neoplasms” [MeSH] or “chronic disease” [MeSH]) or (“tool” or “tests” or “statistical techniques” or “geostatistical techniques”)	
**Inclusion criteria**	**Exclusion criteria**
Published material selected should be in the adopted language for this review which is English	Other languages were not included
Published articles must answer the research questions	Papers not clearly answering any of the suggested research questions
Material used should be selected from the chosen sources only. Within the chosen period: published during the last 15 years	Any material not within the time frame of the study
Original research, clinical trials, systematic reviews are included	Any other type of research

**Table 2:** Main functions and processes of the toolkit.

	Functions and Processes		
	Data management and Adjustment Pre-Processes	Mapping Data	Spatial/ Spatio-Temporal Analysis
**1**	“Cleaning” of a database/double records, etc.	Visualization modeling (spatial and/or temporal dimensions to epidemiologic and other data)	Identifying relationships, clusters and hot spots
**2**	Linking or integrating data	Creating maps, videos, interactive maps and animation with real x,y,z dimensions	Mapping patterns and trends
**3**	Editing several types of data and adopting geographical principles/Locating, geo-referencing and recognizing all types of data (health, social, environmental, satellite images/aerial photos and other quantitative or qualitative data)	Exporting descriptive statistics in the form of graphs or maps (spatial mean and median, standard distance/ellipse, central mean, etc.)	Predicting future patterns
**4**	Estimating epidemiological rates, ratios, indexes etc. (e.g., prevalence, incidence, standardized mortality ratios, etc.) and adjusting data (according to variables that affect the outcomes; e.g., age, sex, socioeconomic status)		Identifying new (optimum) locations
**5**			Identifying risk areas and factors

## Data Availability

Not applicable.

## Supplementary Materials

The following supporting information can be downloaded at: https://www.mdpi.com/article/10.3390/ijerph191912765/s1, Table S1: Steps and statistical tests per process for each type of scenario.
