# Supplementary material for: Evidence-Based Conceptual Collection of Methods for Spatial Epidemiology and Analysis to Enhance Cancer Surveillance and Public Health
Source: Int J Environ Res Public Health. 2022 Oct 6;19(19):12765. doi: 10.3390/ijerph191912765 (PMC9566360; doi:10.3390/ijerph191912765)
Supplement: Supplementary file 1 [file ijerph-19-12765-s001.zip › ijerph-1923245-supplementary.pdf]

Table S1. Steps and statistical tests per process for each type of scenario.

		**Applicability per scenario (A,B,C)**
**Function A**	**Data management and adjustment pre-processes**	
Process 1	**"Cleaning" of a database/double records, etc.**	A,B,C
Steps	**Selection**	
	- Select your dataset (big-data, pooled data etc.).<br>- Describe your dataset in details (source, extent, single or combined dataset, type of cancer eg. low or high prevalence etc.).	
	**Parsing**	
	- Detect any syntax errors. A parser decides whether a string of data is acceptable within the allowed data specification.	
	**Data transformation**	
	- Map the data from its given format into the format expected by the appropriate application. This includes value conversions or translation functions, as well as normalizing numeric values to conform to minimum and maximum values.	
	**Pre-processing**	
	- Clean your dataset from multiple entries, missing values and noise.	
	**Transformation**	
	- Convert (if necessary) the data values from the data format of the cancer registry database to the destination data system (code generation, geo-reference, etc).	
	**Data mining**	
	- Detect any extremity of the data (outlier/change/deviation detection).	
	**Statistical methods for pre-analysis**	
	- Analyze the data using the values of mean, standard deviation, range, or clustering algorithms, it is possible for an expert to find values that are unexpected and thus erroneous. Although the correction of such data is difficult since the true value is not known, it can be resolved by setting the values to an average or other statistical value. Statistical methods can also be used to handle missing values in the previous step, which can be replaced by one or more plausible values, which are usually obtained by extensive data augmentation algorithms.	
Process 2	**Linking or integrating data**	A,B,C
Steps	**Bring datasets on a similar format and file type**	
	**Use software's commands (e.g. join in GIS)**<br>- There are two basic types of joins in GIS tables and geo-data: spatial and relational (based on table IDs).<br>- Spatial join is the key concept in GIS and that is what sets all GIS technologies apart from other relational databases.	
Process 3	**Locating, geo-referencing, editing and recognizing all types of data** (health, social, environmental, satellite images/aerial photos and other quantitative or qualitative data).	A,B,C
Steps	**Import existing digital data in the GIS software**<br>- Direct import of digital data is in most cases the easiest form of digital spatial data conversion.<br>- All software systems provide links to other formats, but the number and functionality of import routines varies between packages.<br>- High-end systems are more likely to provide import functions for a large number of exchange formats. It is also more likely that other data producers will be able to provide GIS data in the native format of the GIS package.	
	**Create new entities** (e.g. locate cancer data per place of residence)<br>-Use the editing tool and locate cancer cases (x,y coordinates).	
	**Add any other type of materials** (e.g. photos, images from satellites).	

	**Geo-reference and recognize all types of data**	
Process 4	**Estimating epidemiological rates, ratios, indexes** etc (e.g. Prevalence, incidence, standardized mortality ratios, etc.) and adjusting data (according to variables that affect the outcomes; e.g. age, sex, socioeconomic).	A,B,C
Steps	**Adjusting data** (according to variables that affect the outcomes; e.g. age, sex, socioeconomic).	
	**Calculate incidence or mortality rates**<br>-Use standardization techniques to manage any population differences and adjust to age or sex or socioeconomic status (direct or indirect standardization).	
**Function B**	**Mapping data**	
Process 1	**Visualization modeling** (spatial and/or temporal dimensions to epidemiologic and other data).	
Steps	**Choose whether the representation of cancer data will be in a viewable medium or format**. In GIS, visualization is used to organize spatial data and related information into layers that can be analyzed or displayed as maps, three-dimensional scenes, summary charts, tables, time-based views, and schematics.<br>-Decide if you will visualize your data spatially or temporally too.	A,B,C
	**Aggregate cancer data if needed** (e.g. for privacy reasons or analysis purposes).<br>-Use distance-weighted averaging such as kernel densities<br>-Use the adaptive buffering	 <br>A,B,C<br>B
Process 2	**Creating maps, videos, interactive maps and animation with real x,y,z dimensions**	
Steps	**Map cancer data using points**	B,C
	**Map cancer data using polygons** (per municipality or state or other regional level).	A,B,C
	**Map cancer data using lines** (e.g to illustrate cancer patient flows).	B,C
Process 3	**Exporting descriptive statistics in the form of graphs or maps** (spatial mean and median, standard distance/ellipse, central mean, etc.).	
Steps	**Identify the spatial mean and median, standard distance/ellipse, central mean**	A,B,C
	**Measure spatial tendency**<br>-The coordinate-wise mean of a point set is the centroid, which solves the same variation problem in the plane (or higher-dimensional Euclidean space) that the familiar average solves on the real line — that is, the centroid has the smallest possible average squared distance to all points in the dataset.	A,B,C
	**Measure spatial dispersion to capture the degree to which points in a point set are separated from each other.**<br>-Several simple measures of spatial dispersion for a point set can be defined using the covariance matrix of the coordinates of the points. The trace, the determinant, and the largest eigenvalue of the covariance matrix can be used.<br>-A measure of spatial dispersion that is not based on the covariance matrix is the average distance between nearest neighbors.	A,B,C
	**Measure spatial autocorrelation to assess and analyze the degree of dependency among observations in a geographic space.**<br>-Use the Moran's Index<br>-Use the Geary's C<br>-Use the Getis's G<br>-Use the local Moran's Index	 <br>B,C<br>A,B,C<br>A,B,C<br>A,B,C
	**Measure spatial homogeneity**<br>-A simple probability model for spatially homogeneous points is the Poisson process in the plane with constant intensity function and the Ripley's K and L functions.	B,C
	**Measure spatial stratified heterogeneity** to explore whether the within-	B,C

	strata variance is less than the between strata-variance. This reflects the essence of nature, implies potential distinct mechanisms by strata, suggests possible determinants of the observed process, allows the representativeness of observations of the earth, and enforces the applicability of statistical inferences.<br>-Measure it by geographical detector q-statistic	
	**Perform spatial interpolation techniques** to estimate the variables at unobserved locations in geographic space based on the values at observed locations.<br>-Use the inverse distance weighting (his attenuates the variable with decreasing proximity from the observed location).<br>-Use the Kriging (this interpolates across space according to a spatial lag relationship that has both systematic and random components. It can accommodate a wide range of spatial relationships for the hidden values between observed locations. Kriging provides optimal estimates given the hypothesized lag relationship, and error estimates can be mapped to determine if spatial patterns exist).	A,B,C
Function C	**Spatial/spatio-temporal analysis**	
Process 1	**Identifying relationships, clusters and hot spots**	
Steps	**Use global clustering tests** to evaluate and identify cancer clusters without pinpointing the specific locations of clusters.<br>- Use the Diggle and Chetwynd's bivariate K-function, Mantel-Bailar's test, Potthoff-Whittinghill method (PW).<br>-Use the Moran's I statistic, Cuzick and Edwards's nearest neighbors<br>- Tango's maximized excess events tests (MEET).	 <br>A,B,C<br><br>B,C<br>A,B,C
	**Use local clustering tests** for specific small-scale clusters and focused cancer clustering.<br>- Use the Anselin's local (hot spots analysis), using the spatial association (LISA)<br>- Use the Besag-Newell test<br>-Use the Kulldorff's spatial test<br>-Use Diggle's test or Stone's conditional test	 <br>A,B<br><br>B<br>A,B,C<br>B,C
	**Use space-time clustering tests** to evaluate and identify cancer clusters in spatio-temporal datasets.<br>-Use the global space-time Knox technique<br>-Use the Diggle's global space-time K-function<br>-Use the extended Kuldorff's spatial test<br>- Use the dynamic continuous-area space-time (DYCAST) system<br>- Use the generalized Bayesian maximum entropy (GBME)	 B<br>A,B,C<br>A,B<br>B,C<br><br>B
Process 2	**Mapping patterns and trends**	
Steps	**Create choropleth maps** to thematically group regions within an area in proportion to the measurement of the statistical variable being displayed on the map (eg. cancer mortality).	A,B
	**Simulate and model disease** (Complex adaptive systems theory as applied to spatial analysis suggests that simple interactions among proximal entities can lead to intricate, persistent and functional spatial entities at aggregate levels).<br>-Cellular automata modeling imposes a fixed spatial framework<br>-Agent-based modeling uses entities (agents) that have purposeful behavior and react, interact and modify their environment while seeking outcomes.	 <br><br><br>A,B<br>B,C
	**Explore spatial interactions**<br>-Use gravity models to estimate the flow of people or the roots of disease or the flows or new cancer cases.<br>-Use computational methods such as artificial neural networks to identify spatial interaction relationships among locations by limiting noise in cancer data and	 B,C<br><br>A,B,C

	increasing the data quality.	
	**Perform trend analysis** (3-dimensional perspective of data using second-order polynomials).	A,B,C
Process 3	**Predicting future patterns**	
Steps	**Use cokriging** to produce prediction, probability, quantile, standard error, and standard error of indicators maps under the same conditions as for the other kriging methods.	B,C
	**Use time-series models**	B,C
	**Use Bayesian projecting techniques**	A,B,C
Process 4	**Identifying new (optimum) locations**	
Steps	**Use multi-criteria analysis** (use model builder in GIS)	A,B,C
	**Use buffering techniques and other geo-analyst tools**	A,B,C
Process 5	**Identifying risk areas and factors**	
Steps	**Use the multivariable spatial regression** (Either frequentist spatial regression or Bayesian spatial regression).	B,C
	**Use spatial autoregressive models**<br>- Use a simultaneous autoregressive (SAR) model<br>- Use the geographically weighted regression (GWR)	 B,C<br>A,B,C
	**Use the Bayesian regression models**<br>-Use the Besag York and Molliè (BYM) model (robust enough and includes fixed covariates along with random effects).<br>-Use the multivariate CAR models for joint spatial modeling of bivariate and multivariate diseases with common risk factors.<br>-Use a combination of models: Bayesian hierarchical modeling in conjunction with Markov Chain Monte Carlo (MCMC) are effective in modeling complex relationships using Poisson-Gamma-CAR, Poisson-lognormal-SAR, or Over dispersed logit models.<br>-Use stochastic /Gaussian processes.	 A,B,C<br>B,C<br>A,B,C<br>B,C
